# Identifying Bacterial and Host Factors Involved in the Interaction of *Mycobacterium bovis* with the Bovine Innate Immune Cells

**DOI:** 10.3389/fimmu.2021.674643

**Published:** 2021-07-15

**Authors:** Federico Carlos Blanco, María José Gravisaco, María Mercedes Bigi, Elizabeth Andrea García, Cecilia Marquez, Mike McNeil, Mary Jackson, Fabiana Bigi

**Affiliations:** ^1^ (Instituto de Biotecnología, Instituto Nacional de Tecnología Agropecuaria) Institute of Biotechnology, National Institute of Agricultural Technology (INTA), Buenos Aires, Argentina; ^2^ (Consejo Nacional de Investigaciones Científicas y Tecnológicas) National Scientific and Technical Research Council (CONICET), Buenos Aires, Argentina; ^3^ (Facultad de Agronomía, Universidad de Buenos Aires) School of Agronomy, University of Buenos Aires (UBA), Buenos Aires, Argentina; ^4^ High Technology Analytical Centre, Laboratory, Buenos Aires, Argentina; ^5^ Department of Microbiology, Immunology and Pathology, Colorado State University, Fort Collins, CO, United States

**Keywords:** innate immune response, *Mycobacterium bovis*, NK cells, ESAT-6, bovine tuberculosis

## Abstract

Bovine tuberculosis is an important animal and zoonotic disease caused by *Mycobacterium bovis*. The innate immune response is the first line of defense against pathogens and is also crucial for the development of an efficient adaptive immune response. In this study we used an *in vitro* co-culture model of antigen presenting cells (APC) and autologous lymphocytes derived from peripheral blood mononuclear cells to identify the cell populations and immune mediators that participate in the development of an efficient innate response capable of controlling the intracellular replication of *M. bovis*. After *M. bovis* infection, bovine immune cell cultures displayed upregulated levels of iNOS, IL-22 and IFN-γ and the induction of the innate immune response was dependent on the presence of differentiated APC. Among the analyzed *M. bovis* isolates, only a live virulent *M. bovis* isolate induced an efficient innate immune response, which was increased upon stimulation of cell co-cultures with the *M. bovis* culture supernatant. Moreover, we demonstrated that an allelic variation of the early secreted protein ESAT-6 (ESAT6 T63A) expressed in the virulent strain is involved in this increased innate immune response. These results highlight the relevance of the compounds secreted by live *M. bovis* as well as the variability among the assessed *M. bovis* strains to induce an efficient innate immune response.

## Introduction

Bovine tuberculosis (bTB) is an important animal and zoonotic disease that causes significant financial loss worldwide and represents a public health hazard. According to the OIE (Office International des Epizooties-World Organisation for Animal Health), out of 188 countries and territories, 82 countries (44%) reported the presence of the disease (https://www.oie.int/es/enfermedad/tuberculosis-bovina/). Losses due to TB are estimated to be US$ 3 billion per year, with more than 50 million cattle infected (1). The costs of this disease are related to reduced productivity in severely affected animals, testing, slaughter of affected animals, movement controls, and trade restriction.

*Mycobacterium bovis* (*M. bovis*), the major causative agent of bTB, is closely related to *Mycobacterium tuberculosis* (*M. tuberculosis*), the causative agent of human tuberculosis (TB). In fact, bTB and TB are clinically similar; both diseases produce granuloma in lungs and lymph nodes, in which lymphocytes and plasma cells surround the infected macrophages. However, in comparison to TB infection, which is mostly arrested at early stages with no or low bacterial replication, cattle *M. bovis* infections progress to more severe stages, producing acute infections with active mycobacterial replication (2). The experimental evidence suggests that species-specific mycobacterial factors significantly contribute to the observed differences in the pathology of TB and bTB (2).

The innate immune system is the first line of host defense against pathogens and therefore plays an important role during the early phase of infection. A relatively large proportion (20-25%) of individuals who are exposed to TB, remarkably, never develops any sign of an immunological memory against *M. tuberculosis*. This suggests that the high efficacy of the innate immune response in these individuals precludes the need of an adaptive immunity. This process is referred to as ‘early clearance’ and can be explained by the presence of a highly effective innate immunity (3).

The most relevant cellular players in the bovine innate immune response against *M. bovis* are antigen presenting cells (APCs) (1, 4), mainly dendritic cells (DCs) and macrophages, gamma delta T (γδ T) cells (5–7) as well as natural killer (NK) cells (8).

Macrophages respond to mycobacterial infection with the production of chemokines and cytokines. These immune mediators trigger innate microbicidal activity and promote the adaptive Th1 immune response, which is characterized by the release of IFN-γ (9). IFN-γ enhances both the microbicidal activity in infected macrophages and the presentation of mycobacterial antigens to T cells (CD4 and CD8) (9).

Macrophages have not only the ability to rapidly respond to TB or bTB infections, but also to induce nonspecific immune memory, which is called trained immunity. Particularly, *M. bovis* might induce trained innate immunity in cattle trough epigenetic regulation of signaling pathway that control the expression of pro-inflammatory cytokines such as IL-1b, type I IFN, IL-6 and TNF-α (10).

γδ T cells play roles in both responses, innate and adaptive, and thus connect the two arms of the immune system. This cell population produces cytokines and participates in direct cytotoxicity as well as in antigen presentation (11, 12). Kennedy et al. have studied cell populations involved in the innate response, particularly γδ T cells, in vaccination trials and experimental infections in cattle (6, 7). On the other hand, bovine activated γδ T cells may upregulate the expression of MHC II, CD80 and CD86, and directly induce CD4 T cell proliferation (13, 14). In addition, γδ T cells are key factors in bTB pathology as they play a crucial role in the granuloma formation (10). NK cells also participate in the linking of innate and adaptive immune responses. For example, in draining lymph nodes NK cells contribute to the early innate response to bacterial infections through the production of IFN-γ and the subsequent development of Th1 immune responses. These NK effector responses against *M. bovis* depend on the interaction with APCs, particularly with DC (8). Siddiqui and Hope have proposed that upon *M. bovis* infection DCs secrete chemokines to induce the recruitment of effector cells, such as NK. Then, the co-localization of NK and DC would enable reciprocal interactions with enhanced secretion of IFN-γ (8). In addition, NK cells display cytotoxic activity and reduce the bacterial count of *M. bovis* BCG-infected macrophages (15).

Certainly, the magnitude of the innate immune response relies on the presence or not of *M. bovis* specific components. For instance, APCs interact with lipid components of the mycobacterial cell wall (16). Furthermore, different *M. bovis* strains or genotypes have shown to induce differential bovine macrophage responses in *in vitro* infections (17, 18).

Although some progress has been made, little is known about the impact of strain variability on innate response mechanisms against bTB and the mycobacterial components necessary to achieve an effective innate immune response. This issue is particularly relevant in the TB vaccinology field, since the development of an efficient adaptive immune response after a vaccination depends, in part, on the first encounter between the vaccine and the innate immune system. On the other hand, the fact that different sub-strains of BCG induce variable protection against *M. tuberculosis* in mice (19) highlights the relevance of the genetic background for live vaccine strategies. Altogether, these concepts indicate that *M. bovis* strain selection is a crucial step for a rational design of an effective live vaccine against bTB.

In this study, we evaluated the innate immune response elicited by three isolates of *M. bovis*, Mb534, MbNCTC10772 and Mb04-303, by using a co-culture model of differentiated APCs and autologous lymphocytes. These *M. bovis* strains have showed different degrees of virulence in animal models of bTB, being Mb04-303 the most virulent, MbNCTC10772 moderately virulent and Mb534 attenuated (20–22).

The results of this study shed light on host and mycobacterial factors involved in the mechanisms of innate immunity capable of controlling the replication of *M. bovis* inside macrophages. The knowledge of these factors is essential for the rational design of effective vaccines against bTB.

## Material and Methods

### Mycobacterial Cultures, Lyophilized Supernatants and Lipid Fractions

Mb534 and Mb04-303 are Argentine isolates obtained from cattle and a wild boar, with tuberculous lesions, respectively. Both *M. bovis* isolates are spoligotype 34, which is the most frequent in Argentina (23). In a mouse progressive TB model, Mb534 was the most attenuated isolate, whereas Mb04-303 was the most virulent and produced extensive pulmonary pneumonia and high bacillary burden (21). Mb04-303 and MbNCTC10772 (a US isolate of *M. bovis* from the UK National Collection of Type Culture, ATCC 19210) were previously tested in an experimental bovine infection (24). Only animals infected with Mb04-303 presented macroscopic tuberculous lesions in lung parenchyma and the associated lymph nodes (bronchial and mediastinal), thus achieving the highest pathology scores of the experiment. On the other hand, few animals infected with MbNCTC10772 showed small macroscopic lesions in head lymph nodes and no tuberculous compatible lesions in lungs.

Based on previous results (20–22) the three strains used in the present study were classified as mildly (Mb534), moderately (MbNCTC10772) and highly (Mb04-303) virulent.

Whole genome sequences of Mb04-303 and Mb534 are available in public database (DDBJ/EMBL/GenBank under accession numbers AVSW01000000 and JQEM00000000, respectively).

Recombinant *M. bovis* strains were previously obtained (25). Briefly, the *esat-6* gene was PCR amplified from Mb534 and Mb04-303 strains and cloned in pVV16 vector (BEI Resources). The recombinant plasmids were used to transform the Mb534 strain.

All isolates were grown at 37°C in Middlebrook 7H9 (BD, USA) liquid medium enriched with 0.4% pyruvic acid and 1% albumin dextrose complex (ADC) with or without 0.5% Tween 80. Bacterial cultures were used at exponential growth phase; bacterial viability was greater than 95%, as determined by BacLight kit (Invitrogen), in all cultures used in this study. The different strains were inactivated at 95°C for 30 min. Lyophilized culture supernatants (CS) were obtained from 30-day cultures (50 mL) grown without Tween 80. The cultures were centrifuged at 2,500 g for 20 min and the supernatants were filtered (pore size: 0.22 μm), frozen in liquid nitrogen and lyophilized in 50-mL conical tubes for 4 days.

*M. bovis* lipids were obtained from CS (150 mL), purified and resolved according to previous publications (26–28).

### Two Hybrid Assays

The mutant variant of *esat-6* gene, which encodes a protein with alanine (A) in position 63 instead of a threonine (T), and the wild type gene were PCR-amplified from Mb04-303 and Mb534, respectively (25). The genes were cloned as fusion to the T25 and T18 fragments of the adenylate cyclase in the BamH1 site of the bait pKT25 and prey pUT18C vectors, respectively (29). The integrity of the cloned genes was confirmed by sequencing. *E. coli* BTH101 was co-transformed with a bait and prey recombinant plasmids. Co-transformants were selected on LB-agar medium containing ampicillin and kanamycin and then cultivated in LB medium containing ampicillin (100 μg/mL) and kanamycin (50 μg/mL) at 30°C with shaking.

The interaction of fusion proteins encoded in T18-Zip and T25-Zip plasmids was used as a positive control.

The β-galactosidase activity was assessed by pelleting 1 mL of recombinant *E. coli* cultures (cultured at 30°C for 5 days) and measuring according to the procedure described by Miller (30).

### Animals and Sample Collection

Healthy adult calves (36-60 months of age) were sampled from a herd settled in an INTA field with no history of bTB and paratuberculosis within the past 5 years. The animals were breed cross calves (Hereford and Aberdeen Angus) susceptible to bTB and were negative for IFN-γ ELISA assay (Bovigam) as well as to tuberculin skin test (data not shown) against PPDb and PPDa (Purified Protein Derivatives from *M. bovis* and Purified Protein Derivatives from *Mycobacterium avium*, respectively). Sample collection and animal handling were done in compliance with the regulations of the Ethical Committee of INTA (CICUAE).

### Mononuclear Cell Preparation, Macrophage Differentiation and Co-Cultures

Heparinized blood (20 mL) from each animal was used for peripheral blood mononuclear cells (PBMC) isolation by gradient centrifugation over Histopaque 1077 (Sigma-Aldrich), following the manufacturer’s protocol. PBMC were incubated at 37°C in RPMI complete medium (Invitrogen) supplemented with 10% autologous plasma and 1x Antibiotic-Antimycotic (Anti Anti) (Invitrogen) in T25 tissue culture flask for RNA extraction or 24-well tissue culture plates for flow cytometry determinations. PBMC were seeded at 1x10^7^ or 2.5-3x10^6^ in tissue culture flasks or in each well in culture plates, respectively. After 24 h of incubation, non-adherent cells were removed and cultured separately. Adherent cells were incubated with 10% autologous plasma for 4-5 days until macrophage differentiation. After this incubation period, more than 93% of the adherent cells were CD14+ (**Figure S1**).

Primary culture macrophages or PBMC were infected for 3-4 h with Mb04-303 (viable or heat inactivated), MbNCTC10772 or Mb534 at a multiplicity of infection (MOI) of 1 for flow cytometry determinations and mycobacterial intracellular replication assays or MOI of 5 for transcription analysis. Cell monolayers were washed three times with PBS to eliminate any extracellular bacterium and then co-cultured for another 16 h or 24 h with the autologous lymphocytes (1:10) for transcription analysis (16 h) or flow cytometry determinations (24 h). Alternatively, PBMC were isolated, seeded in culture plates at 2.5-3x10^6^ and infected with Mb04-303 (viable or heat inactivated) at MOI of 1 in relation to monocyte percentages among total PBMC (10%) in order to perform infections in absence of differentiated macrophages.

For the mycobacterial intracellular replication assay, macrophages from three different calves were infected in 24-well tissue culture plates with the different strains in triplicate for 3 h. Then, the wells were washed three times with PBS and the infected macrophages were incubated for 72 h with or without autologous lymphocytes. Cells were lysed with triton X-100 and seeded in 7H10-pyruvate medium supplemented with ADC (Sigma-Aldrich). Colony forming units (CFUs) were counted after 21 days for all the evaluated experimental conditions.

### Viability Assays

Macrophage viability was assessed in non-infected and infected cultures. Cell viability was also studied in infected/stimulated lymphocytes and in macrophages infections using Fixable Viability Dye eFluor 660 (eBioscience). Briefly, macrophages from three different animals were detached using cold PBS-EDTA 20 μM and washed twice with PBS. Non-adherent cells were directly washed with PBS. Resuspended cells were stained following the manufacturer’s instructions. Viability was studied in 20,000 events using FACScalibur BD flow cytometry in each condition.

### Transcriptional Analysis

Briefly, non-adherent cells of the infected co-cultures described above were centrifuged at 362 xg for 10 min and the pellets were treated with 1 mL Trizol (Invitrogen). This Trizol suspension was used to detach the autologous macrophage monolayers and total RNA was obtained from the complete co-cultures following the manufacturer’s protocol. Total RNA quality and quantity were estimated by UV spectrophotometry (Nanodrop) and electrophoresis on 0.8% agarose gel. DNA-free RNA (1µg) was mixed with 50 ng random primers (Invitrogen) in 20 µl final volume and reverse transcribed to total cDNA with SuperScript II (Invitrogen) following the manufacturer’s instructions. Each real time quantitative PCR (qPCR) reaction was performed using 1 µl of a two-fold dilution of the cDNA as template.

All primers were designed using Primer3 (http://bioinfo.ut.ee/primer3-0.4.0/) (**Table S1**). The qPCR reactions were performed with a 20 µL final volume mix of Taq platinum (Invitrogen), SYBR Green I (0.002X) (Invitrogen), ROX (0.02X) (Invitrogen) and dNTPs (4 µM) (Invitrogen) on Applied Biosystem Step One Plus using standard cycling conditions. All reactions were performed in duplicate and qPCR data were analyzed using the 2 ^–ΔΔCT^ with efficiency correction as described previously (18). For each animal, the untreated condition and *gadph* were used as the calibrator and reference gene, respectively. Final calculations and a paired nonparametric test were performed using Fg statistical software (https://www.infostat.com.ar/index.php?mod=page&id=34&lang=en).

### Flow Cytometry

Each flow cytometry determination was performed using 2x10^6^ cells from seven or five animals. The expression of WC1 (MCA1655F), CD335 (NKp46) (MCA2365A488), CD25 (MCA2430F and MCA2430PE), and IFN-γ (MCA1783PE) were assessed by staining cells with different fluorescent-conjugated monoclonal antibodies (AdD Serotec). For each FACS experiment, tubes with the corresponding isotype controls were set (MCA929F, MCA929PE, MCA928A488, MCA929A647). Briefly, for the surface markers study, cells were washed twice with PBS and 4% foetal bovine serum (FBS) (Invitrogen), stained with the specific antibodies and finally fixed with 4% paraformaldehyde (PFA) (Sigma-Aldrich). Because antibodies against WC1 and CD335 surface markers carry fluorochromes with very similar excitation and emission spectrum, these surface markers were labelled separately. The stained cells were analyzed in a FACScalibur cytometer (BD) using Cell Quest software. Analysis gates were set on lymphocytes according to forward and side scatter (**Figure S2**). IL-2R (CD25) expression was analyzed in WC1+ and CD335+ populations as a marker of cell activation.

For IFN-γ analysis, cells were stained intracytoplasmatically using Monensin (1X) (Sigma-Aldrich) as protein transport inhibitor and phorbol myristate acetate and calcium ionophore (PMA-ionomycin) (Sigma-Aldrich) as a positive control, by following standard BD protocols. Percentages of CD25+ and IFN-γ + cells were expressed as the percentage of WC1+ or CD335+ cells positive for these markers among total WC1+ or CD335+ populations. For each labeled sample, 50,000 events from a single cell well were analyzed.

## Results

### Virulent *M. bovis* 04-303 Induces Th1 Cytokines in Naïve Immune Cells

In a previous report, infection of cattle with the strain Mb04-303 produced higher levels of IFN-γ in respond to *in vitro* stimulation with *M. bovis* antigens than infection with the strain MbNCTCT10772 (20). Although Mb534 has never been tested in cattle, in a mouse model this strain was less lethal, and produced limited tissue damage, in relation to Mb04-303 (21).

**Figure 1:**
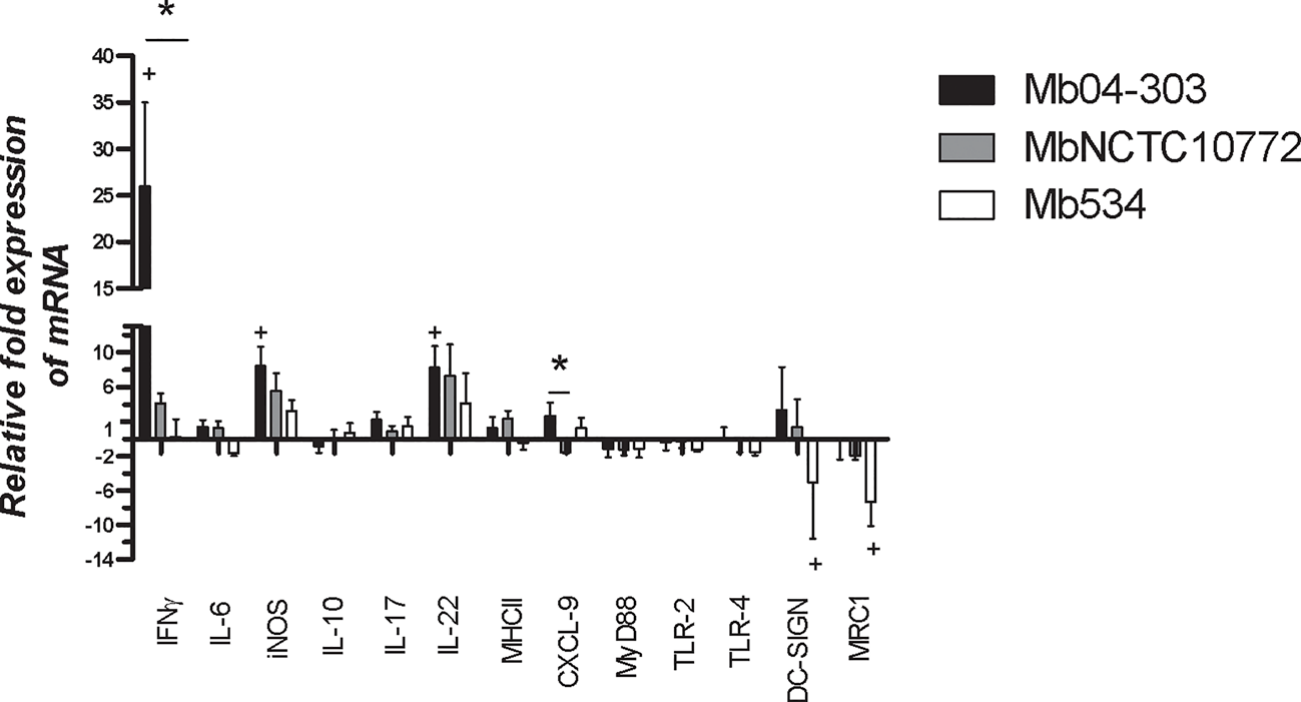
Expression of immune mediators assessed by RT-qPCR in co-cultures from five to seven different calves (N=5-7) infected with *M. bovis* strains (MOI=5). After infection, bovine macrophages were incubated with autologous lymphocytes (1:10) for 16 h. Total RNA was extracted using Trizol standard protocols. Relative expression of each immune mediator was analyzed using *gapdh* as a reference gene and the non-infected condition as calibrator (bars represent mean values and whiskers SEM). + indicates significant upregulation in relation to the non-infected condition (p<0.05) analyzed by pair wise fixed reallocation randomization test (https://www.infostat.com.ar/index.php?mod=page&id=34&lang=en). *indicates significant differences between co-cultures infected with different strains (p<0.05) determined by Friedman test.

We evaluated the expression of immune mediators in co-cultures of *M. bovis*-infected macrophages and autologous lymphocytes. Only strain Mb04-303 induced the transcription of IFN-γ, iNOS and IL-22 genes (p<0.05) over the basal levels of the non-infected co-cultures (**Figure 1**). The expression of these immune mediators was also higher upon MbNCTC10772 infection, but with no significant differences in relation to the non-infected cells. Mb534 infected macrophages maintained basal levels of the expression of these mediators, similar to those of the non-infected cells. IFN-γ also was upregulated in Mb04-303-infected co-cultures in comparison with Mb534. Moreover, CXCL9, an important chemokine and a proposed biomarker of infection (31), showed different expression patterns between Mb04-303 and MbNCTC10772 infections.

To characterize a pro-inflammatory activation pathway and relevant cell receptors, we studied the expression of Toll like receptor 2 (TLR2), Toll like receptor 4 (TLR4), Myeloid differentiation primary response protein (MyD88), dendritic cell–specific intercellular adhesion molecule-3 grabbing nonintegrin (DC-SIGN) and the mannose receptor type 1 (MRC1). The level of expression of TLR2, TLR4 and MyD88 was unaltered upon *M. bovis* infections (**Figure 1**).

DC-SIGN and MRC1 receptors were downregulated only in Mb534 infected co-cultures (p<0.05). These results are consistent with the unaltered expression of pro-inflammatory cytokines and elimination of Mb534 by host cells as described in the *Discussion* section.

### Cell Activation in *M. bovis* Infected Co-Cultures

**Figure 2:**
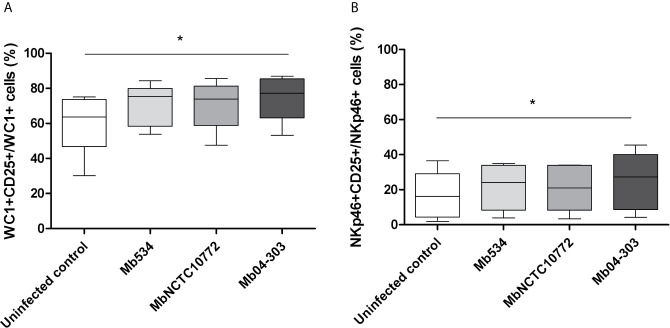
CD25 (IL-2R) expression assessed by flow cytometry in infected co-cultures (MOI=1) of cells obtained from five different calves (N=5). For each animal sample and condition tested a single cell well was analyzed. The results are expressed as CD25+ cells among total WC1+ **(A)** and NKp46+ **(B)** populations. Whiskers and plots indicate minimum, maximum and mean values. *p<0.05 using Friedman test with Dunn’s post- test for multiple comparisons.

To evaluate cell activation in Mb04-303, MbNCTC10772 or Mb534 infected co-cultures, we assessed the IL-2R (CD25) surface expression in NKp46+ and WC1+ cells. Significant upregulation of CD25 for WC1+ and NKp46+ cells was evident after infection with Mb04-303 in comparison to the non-infected condition (p<0.05) (**Figure 2**). MbNCTC10772 and Mb534-infected co-cultures did not show this activation (**Figure 2**). The increased cellular activation in Mb04-303 infected co-cultures is consistent with the pro-inflammatory immune cytokine profile observed in **Figure 1**.

### Innate Immune Response Elicited by Mb04-303 Inhibits Growth of *M. bovis* in Macrophages

**Figure 3:**
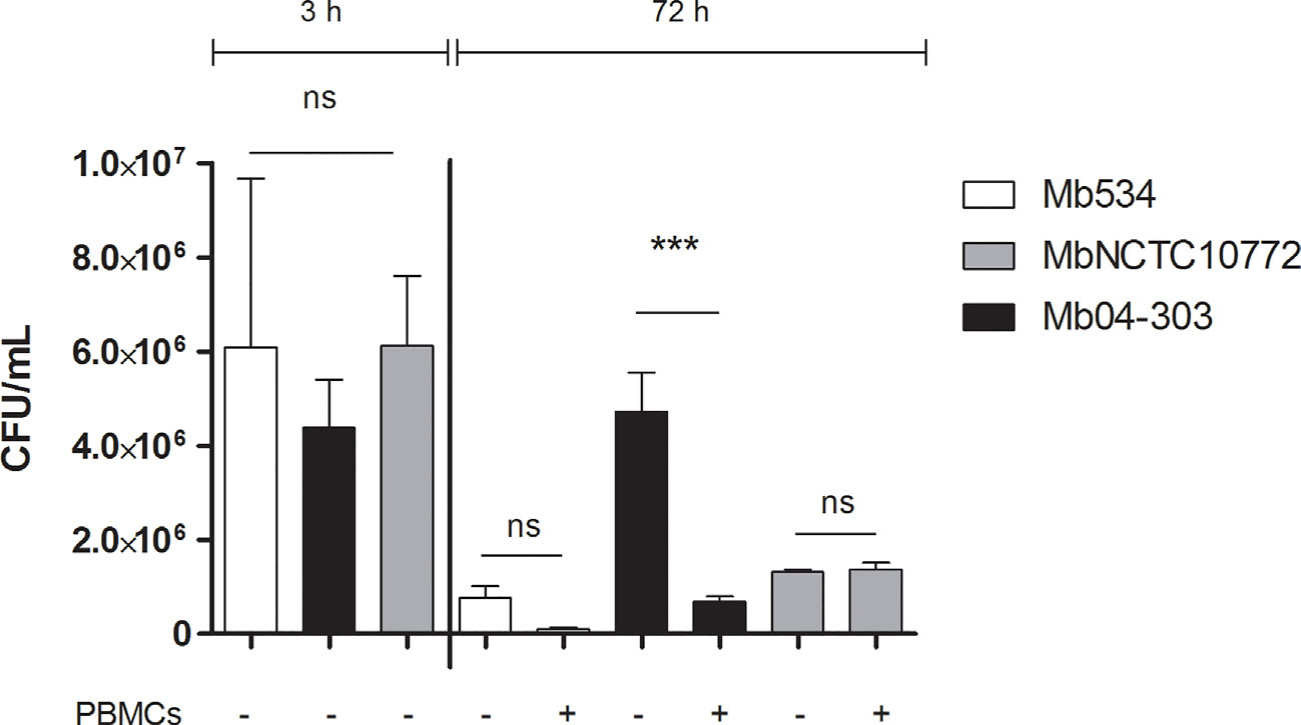
Mycobacterial growth inhibition assay. Macrophages were infected with *M. bovis* strains (MOI=1) and co-cultured with autologous lymphocytes or culture media alone (control condition). Colony forming units were counted 3 h post infection (to detect any variation in inoculum between strains) and at 72 h. Cells were purified from three different calves (N=3). Macrophages and co-cultures were infected in parallel and in triplicate for each condition (technical replicates); bars represent mean values and whiskers SEM. ***p<0.001 ANOVA test with Bonferroni post-test for multiple comparisons. ns, not significant.

We next assessed if the observed innate immune response was capable of inhibiting the *M. bovis* intracellular growth. Infections of differentiated macrophages from three PPDb- and PPDa- cattle with Mb04-303, MbNCTC10772 or Mb534 at MOI=1 showed similar patterns 3 h post infection (T0), as assessed by CFU counts (**Figure 3**). However, only strain Mb04-303 maintained the macrophage infection level after 72 h. Remarkably, CFUs of strain Mb04-303 were higher in infected macrophages than in co-cultures with autologous lymphocytes 72 h post infection. This finding reveals an inhibition of intracellular Mb04-303 replication in co-cultures (p<0.001).

As evidenced by flow cytometry analysis, infected co-cultures conditions displayed a slight, but not significant, increase of cell death in relation to macrophages alone (**Figure S3A**). Thus, the inhibition of Mb04-303 replication in infected co-cultures was not related to increased cell death. No bacterial load reduction was evident in MbNCTC10772 or Mb534 infected co-cultures in comparison with the control condition (macrophages alone). Altogether, these results indicate that the innate immune response capable of reducing the intracellular viability of Mb04-303 depends on non-adherent cells. By contrast, non-adherent immune cells seem to not contribute to the control of MbNCTC10772 or Mb534 infections by macrophages.

### NKp46 and WC1+ Cells Produce IFN-γ in Co-Cultures Infected With Mb04-303

To identify the cell populations responsible for increased IFN-γ transcription, we measured the production of this cytokine in the co-cultures infected with Mb534, MbNCTC10772 or Mb04-303 using flow cytometry and specific antibodies.

**Figure 4:**
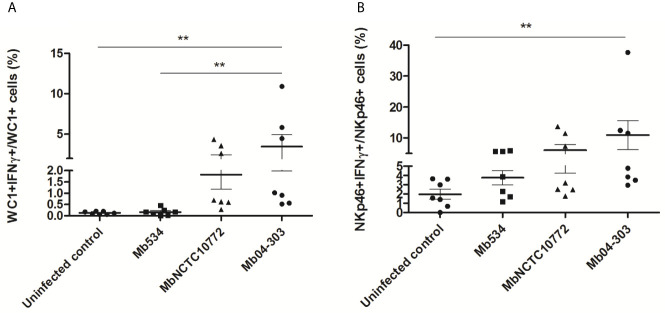
IFN-γ intracytoplasmic quantification assessed by flow cytometry in infected co-cultures (MOI=1) of cells obtained from seven different calves (N=7). For each animal sample and condition tested, a single cell well was analyzed. The results are expressed as IFN-γ+ cells in total WC1+ **(A)** and NKp46+ **(B)** populations. Each symbol represents an individual animal; horizontal lines represent mean values and whiskers SEM. **p < 0.01 determined by Friedman test with Dunn’s post-test for multiple comparisons.

WC1+ and NKp46+ cells in Mb04-303 infected co-cultures produced higher levels of IFN-γ than the non-infected controls (p<0.01) (**Figure 4**). WC1+IFN-γ+ cells were also increased in infections with Mb04-303 relative to infections with the attenuated strain Mb534 (p<0.01) (**Figure 4A**). In agreement with IFN-γ RT-qPCR results (**Figure 1**), no significant IFN-γ increase was apparent in Mb534 and MbNCTC10772 infected co-cultures in comparison with non-infected controls (**Figure 4**). In Mb04-303 infected co-cultures, NKp46+ cells were the most responsive cell population, with a mean of 10.95% NKp46+ IFN-γ+ among total NKp46+ cells (**Figure 4B**). WC1+IFN-γ+ represented 3.46% of the total WC1+ population (**Figure 4A**). Adherent cells, most of them differentiated to macrophages, seemed to express IFN-γ upon infection with *M. bovis* strains, although the results were not significantly different to those of the non-infected cells (data not shown). This result is in agreement with previous reports where macrophages are described as a poor source of IFN-γ compared with NK and T cells (32).

### Upregulation of IFN-γ in Co-Cultures Requires Viable Infecting Bacteria and Mature APCs

To assess the relevance of APCs in the IFN-γ production (**Figure 4**), we infected PBMC with Mb04-303 and quantified NKp46+IFN-γ+ and WC1+IFN-γ+ cells. In this cell population, in which monocytes were not differentiated to mature APCs, Mb04-303 infection did not induce significant IFN-γ production in relation to control conditions (**Table 1**).

**Table 1:** Frequency of cells producing IFN-γ in different cell systems upon stimulation with heat inactivated (HI) or viable bacteria (V).

Cell population	γδ WC1+		NKp46+	
cell culture/treatment	Untreated control	Mb04-303	Untreated control	Mb04-303
Co-culture/V	0.129 (0.023)	3.460 (1.477) p<0.01	1.979 (0.543)	10.950 (4.691) p<0.01
Co-culture/HI	0.796 (0.324)	0.594 (0.312) p>0.05	3.303 (1.429)	4.600 (2,594) p>0.05
PBMC/V	0.554 (0.234)	0.809 (0.255) p>0.05	1.661 (0.656)	2.158 (0.631) p>0.05
PBMC/HI	0.222 (0.077)	0.302 (0.083) p>0.05	0.862 (0.405)	1.530 (0.917) p>0.05

Percentage mean and SEM (between parenthesis) of IFN-γ+ cells determined by flow cytometry in co-cultures or PBMC obtained from five different calves (N=5) treated with heat inactivated bacteria (HI) or viable (V) Mb04-303. For each animal sample and condition tested, a single cell well was analyzed. Mean and SEM values from **Figures 4A, B** are depicted in the first row (Co-culture/V); the rest of the tested conditions did not induced significant IFN-γ upregulation relative to the untreated control (p values from Friedman test are indicated).

To determine whether compounds secreted or produced *in vivo* by *M. bovis* are essential to mount an innate immune response, we used heat-inactivated Mb04-303 to stimulate macrophages co-cultivated with autologous lymphocytes. No significant production of IFN-γ+ cells was detected under this condition. As expected, stimulation of PBMC with heat-inactivated Mb04-303 neither increased IFN-γ+ cell levels compared to the non-stimulated control condition (**Table 1**).

Thus, mature APCs are relevant in IFN-γ expression probably through cell-to-cell contact or secreted immune mediators from mature APCs. The incapacity of inactivated mycobacteria to induce IFN-γ production suggests that the activation of IFN-γ pathway requires secreted bacterial compounds that are not released when the bacteria are dead.

### The Most Virulent *M. bovis* Strains Induced the Lowest Expression Levels of Natural Cytotoxicity Receptor NKp46

**Figure 5:**
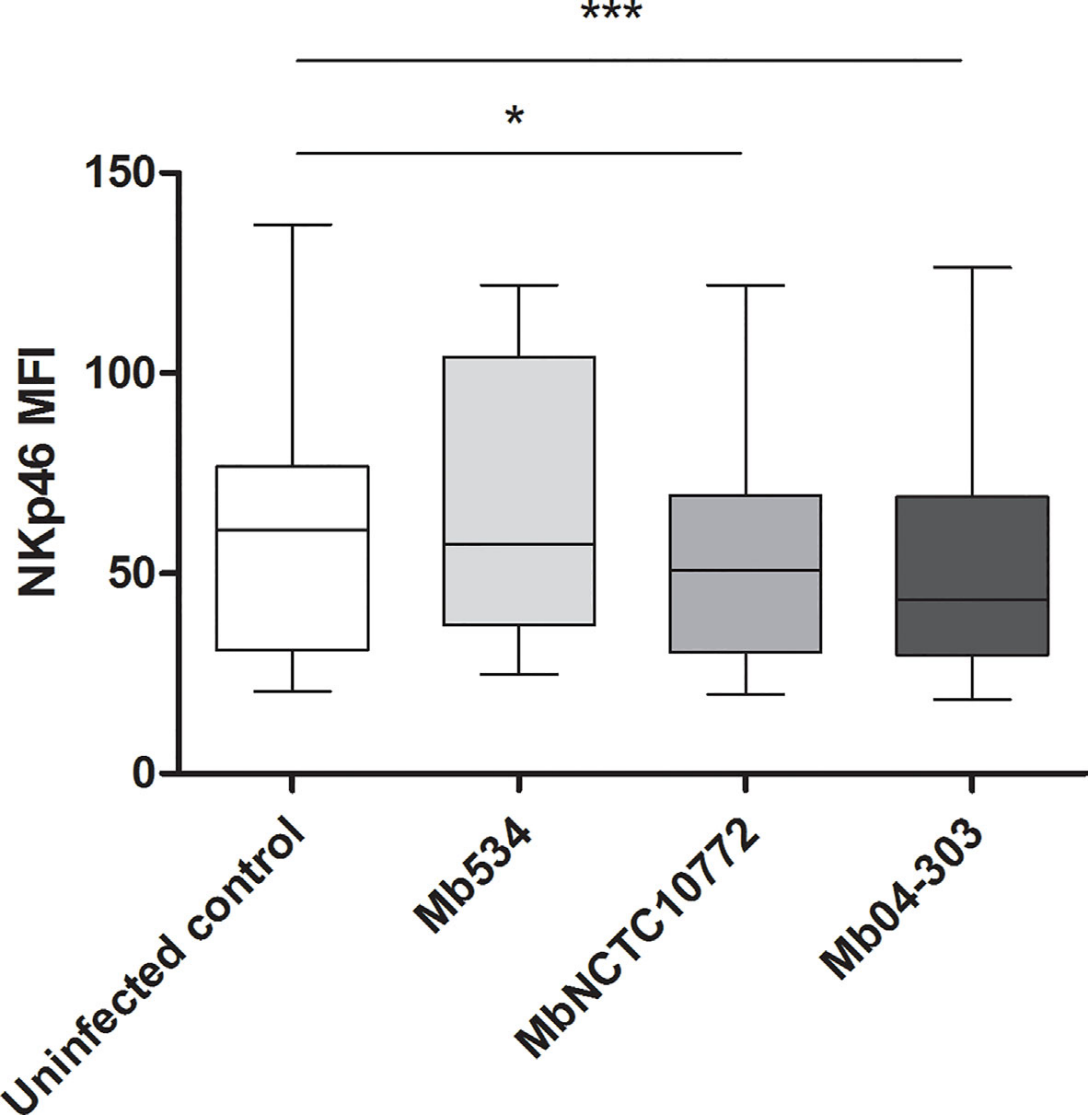
Mean fluorescence intensity (MFI) of NKp46 expression in NK cells. For each animal sample and condition tested a single cell well was analyzed. Whiskers and plots indicate minimum, maximum and mean values (*significant differences; p < 0.05 and ***p < 0.001 by Friedman test).

As a way to analyze the NK cell activation in the *M. bovis* infected co-cultures, we determined the mean fluorescence intensity (MFI) in the NKp46+ population. To study the MFI in histogram plots, we used the data of CD25 and IFN-γ determinations and gated the NKp46+ cells. Mb04-303 and MbNCTC10772 infections induced a downregulation of NKp46+. Moreover, Mb04-303 infections showed the lowest levels of NKp46 expression (p<0.001), followed by MbNCTC10772 infections (p<0.05) (**Figure 5**). Mb534 infections achieved the highest expression of NKp46, although the value was not significantly different from the non-infected controls.

### Secreted Compounds of Mb04-303 Are Relevant for IFN-γ Expression

We hypothesized that the upregulation of IFN-γ in Mb04-303 co-cultures was due to the presence of mycobacterial proteins with acyl or glycosyl groups, lipids or other bacterial secreted factors, and therefore performed studies with lyophilized Mb04-303-CS. Again, IFN-γ production was assessed by flow cytometry in γδ WC1+ and NKp46+ cell populations. For each analyzed animal, we used 10 mg of lyophilized culture medium alone (CS 7H9-P-ADC 10 mg) as a control.

**Figure 6:**
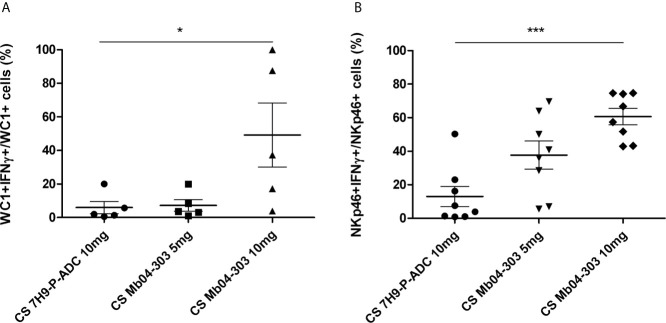
IFN-γ intracytoplasmic quantification by flow cytometry in co-cultures of cells stimulated with 5 or 10 mg of Mb04-303 lyophilized culture supernatants (CS). Lyophilized culture medium alone (CS 7H9-P-ADC 10mg) was used as a negative control. For each animal sample and condition tested, a single cell well was analyzed. Each symbol represents an individual animal; horizontal lines represent mean values and whiskers SEM. The results are expressed as IFN-γ+ cells in total WC1+ **(A)** (N=5) and NKp46+ **(B)** (N=8) populations (*p < 0.05 and ***p < 0.0001 significant differences by Friedman test with Dunn’s post-test for multiple comparisons).

WC1+ and NKp46+ cells responded to the secreted compounds from the bacteria by increasing IFN-γ levels in these cell populations (p<0.05), upon incubation with 10 mg of CS (**Figure 6**). Although cell viability decreased to a mean value of 77.6% (**Figure S3B**) with the highest CS concentration (10 mg/mL), the studied lymphocyte gate was suitable for the analysis. The cell viability decrease may be because of high osmolality of the cell culture media, since pH was unaltered.

Percentages of NKp46+IFN-γ+ cells upon stimulation with CS were higher than those of co-culture infections with viable Mb04-303 [with a mean value of 27.89% (5 mg CS) and 57.41% (10 mg CS) of NKp46+IFN-γ +/total NKp46+ compared to 10.95% of NKp46+IFN-γ +/total NKp46+ cells in co-cultures infected with viable Mb04-303]. Moreover, percentages of NKp46+IFN-γ+ cells increased with higher CS concentrations. For WC1+ cells, only the highest CS concentration (10 mg) induced a significant IFN-γ expression (p<0.05).

Since the bacterial lipids are relevant for the interaction of pathogenic mycobacteria with their host (33), we compared the mycolipid contents in CS of Mb04-303 and Mb534. Thin layer chromatography (TLC) and liquid-chromatography and mass spectrometry (LC-MS) experiments showed that Mb04-303 secreted more mycoside B than Mb534 (**Figure S4**). Mycoside B is a lipid that shares much of the biosynthetic pathways of phthiocerol dimycocerosates (PDIM), PDIMA and PDIMB; all these lipids are involved in the interaction of pathogenic mycobacteria with the host cells (33).

The most distinctive feature of *M. bovis* 04-303 is the non-synonymous mutation in its *esat-6* gene. This gene (also called *esxA*) encodes 6 kDa early secretory antigenic target (ESAT-6). The mutation changes a threonine (T) in position 63 for an alanine (A) (25). To assess the impact of ESAT-6 mutation in the magnitude of the innate immune response induced by the bacilli, we expressed the mutant variant of ESAT-6 (ESAT-6 T63A) in *M. bovis* 534. As control, we overexpressed the wild type version of ESAT-6 in 534.

Both recombinant 534 strains secreted equivalent amounts of ESAT-6 (**Figure S5**). Macrophages co-cultivated with autologous lymphocyte were infected with the recombinant strains. The most responsive cells in the previous determinations were NKp46+, therefore we decided to focus on this cell population to study the innate immune response induced upon infection with Mb534 expressing the different versions of ESAT-6.

**Figure 7:**
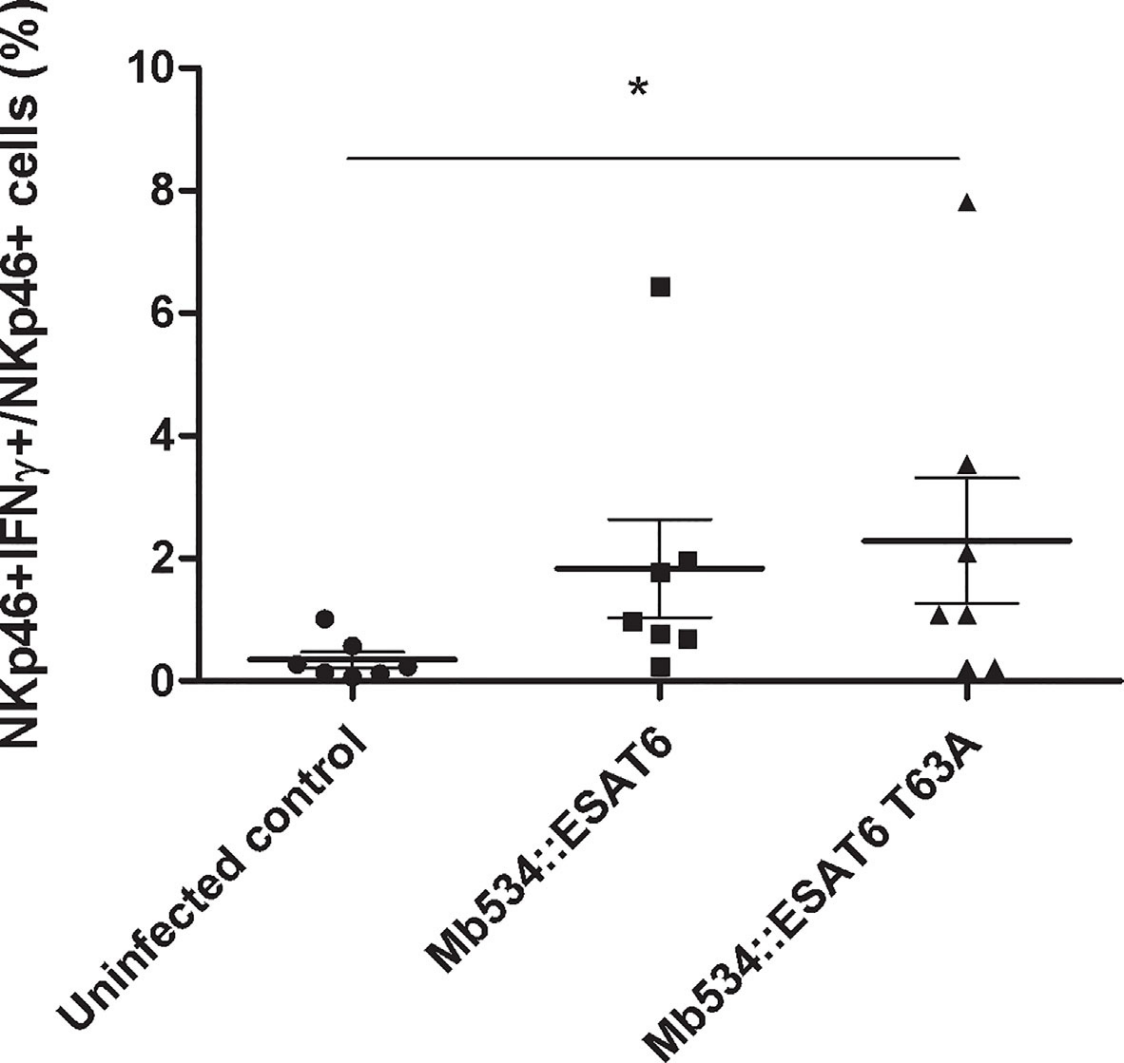
Percentages of NKp46+ IFN-γ+ in co-cultures from seven calves (N=7) infected with the recombinant versions of Mb534 expressing wild type ESAT-6 (Mb534::ESAT6) and the Mb04-303 mutant version of ESAT-6 (Mb534::ESAT6 T63A). For each animal sample and condition tested, a single cell well was analyzed. Each symbol represents an individual animal; horizontal lines represent mean values and whiskers SEM. The results are expressed as IFN-γ+ cells in total NKp46+ population (*significant differences p < 0.05 by Friedman test with Dunn’s post-test for multiple comparisons).

**Figure 8:**
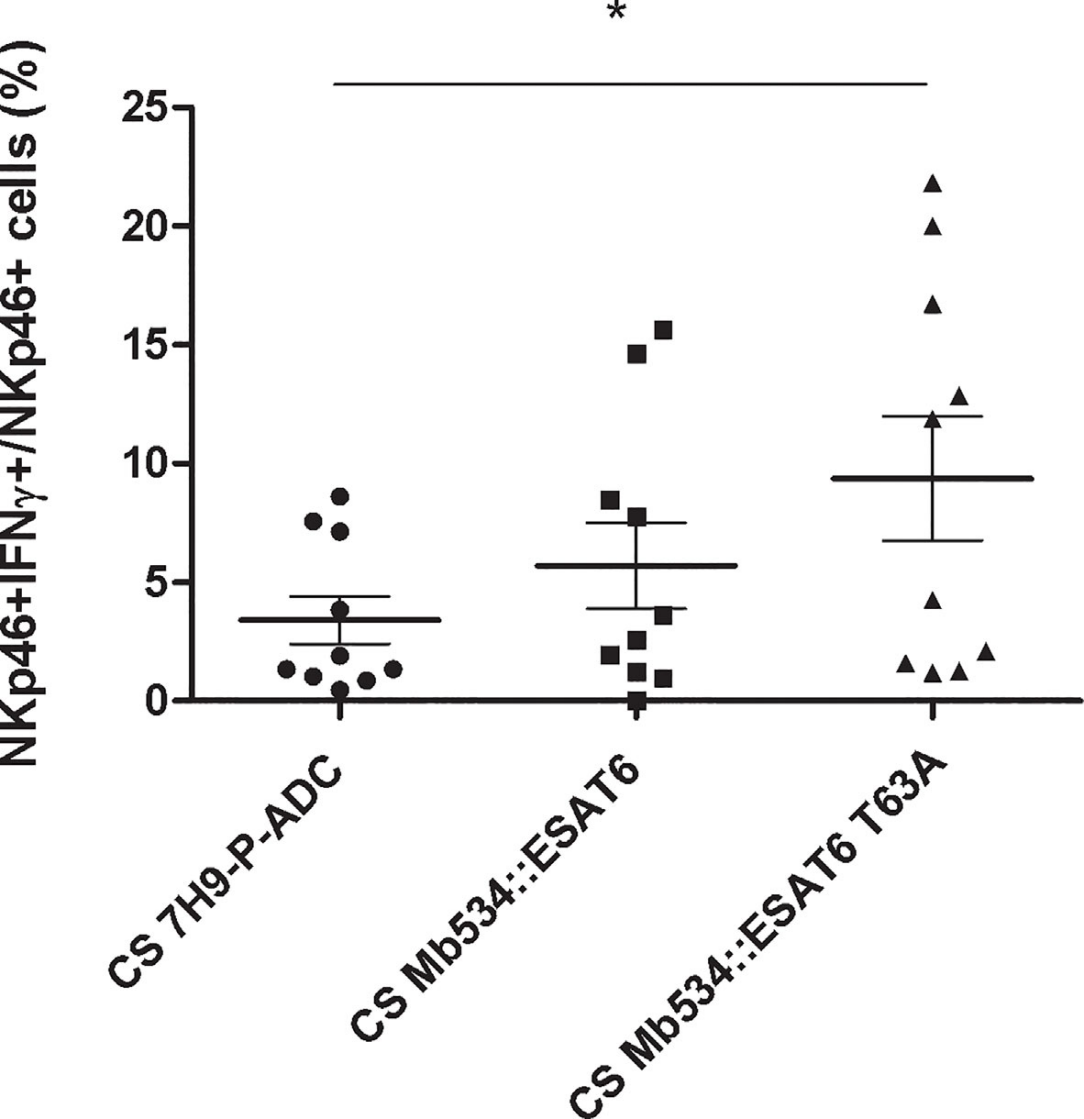
Percentages of NKp46+ IFN-γ+ in co-cultures from ten calves (N=10) stimulated with 10 mg of the lyophilized culture supernatants (CS) from the recombinant versions of Mb534 expressing wild type ESAT-6 (Mb534::ESAT6) and the Mb04-303 mutant version of ESAT-6 (Mb534::ESAT6 T63A). Lyophilized culture medium alone (10 mg) was used as a negative control (CS 7H9-P-ADC). For each animal sample and condition tested, a single cell well was analyzed. Each symbol represents an individual animal; horizontal lines represent mean values and whiskers SEM. The results are expressed as IFN-γ+ cells in total NKp46+ population (*significant differences p < 0.05 by Friedman test with Dunn’s post-test for multiple comparisons).

Only infection with *M. bovis* 534 expressing ESAT-6 T63A (Mb534::ESAT6 T63A) increased the percentage of NKp46+IFNγ+ cells compared with the un-infected condition (**Figure 7**). In addition, cell co-cultures incubated with 10 mg of lyophilized Mb534::ESAT6 T63A-CS induced significant production of IFNγ in NKp46+ cells, in comparison with the un-stimulated condition (**Figure 8**), whereas incubation of these co-cultures with lyophilized Mb534::ESAT6-CS did not stimulate the production of IFNγ in NKp46+ cells.

### Mutation in *esat-6* From Mb04-303 Enhances Dimer Formation

Self-interaction of ESAT-6 and interaction with CFP-10 has been previously demonstrated *in vitro*; moreover, the formation of homo and hetero multimers of these ESX-1 proteins may be crucial in the interaction of *M. tuberculosis* with the host cells (34, 35).

To evaluate the capacity of ESAT-6 and ESAT-6 T63A to form dimers, we used an *E. coli* two-hybrid system. The genes encoding both allele variants of ESAT-6 were cloned into the bait and prey plasmids as fusion to T25 and T18 fragments of the catalytic domain of the adenylate cyclase toxin (CyaA) of *Bordetella pertussis* (29). All different plasmid combinations (pkT25-ESAT-6T63A+pUT18c-ESAT-6T63A, pkT25-ESAT- 6+pUT18c-ESAT-6, pkT25-ESAT-6+pUT18c-ESAT-6T63A, pkT25-ESAT-6 T63A+pUT18c-ESAT-6 and control plasmids) were co-transformed in *E. coli* BTH.

**Figure 9:**
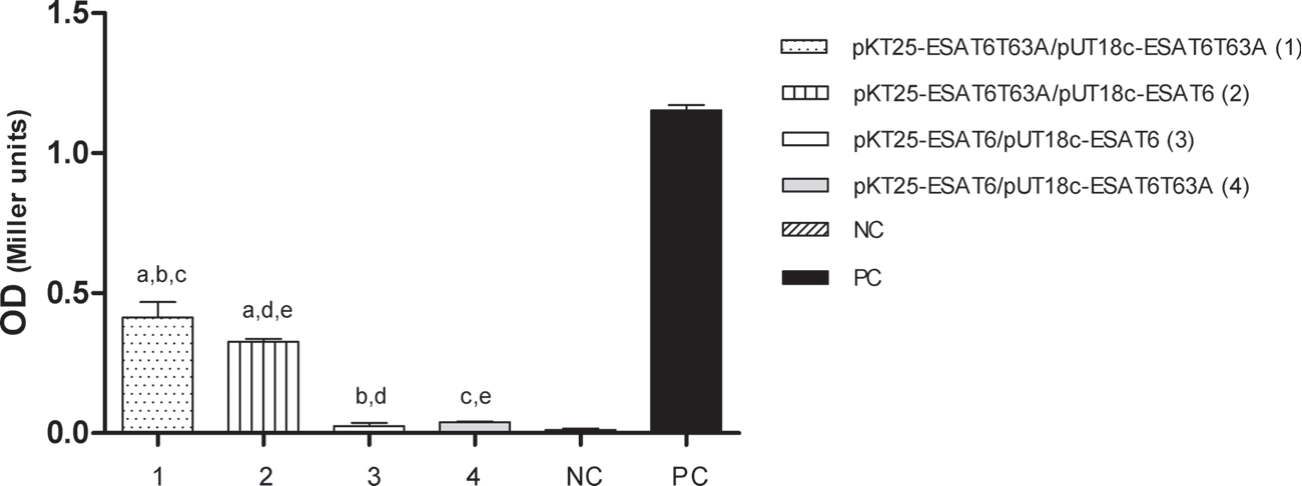
*In vivo* interaction of ESAT-6 alleles. Protein interactions were determined by using a bacterial two- hybrid assay. *E. coli* BTH 101 cells were transformed with the plasmids described in the figure and the β-galactosidase activity was measured as described in M&M. The bars represent β-galactosidase activity, expressed as Miller units [A420x1000/reaction time (min)xA600] ± S.D. of triplicate. Plasmids pUT18c-ESAT-6 and pkT25-ESAT-6 are respectively pUT18c and pKT25 vectors carrying ESAT-6 WT. Plasmids pUT18c-ESAT-6T63A, and pkT25-ESAT-6T63A are respectively pUT18c and pKT25 vectors carrying ESAT-6T63A. NC, negative control; PC, positive control. (Statistical analysis was performed using ANOVA and Bonferroni post test to compare among the different plasmid constructions). a: significant difference between 1 and 2 (p<0.05); b: 1 and 3 (p<0.0001); c: 1 and 4 (p<0.0001); d: 2 and 3 (p<0.0001) and e: 2 and 4 (p<0.0001).

Adenylate cyclase activity was determined in *E. coli* transformants by measuring LacZ activity (**Figure 9**) and by growing the bacteria in medium containing maltose as unique carbon source (**Figure S6**). No protein interaction was detected in co-tranformants expressing ESAT-6 wild type as fusion to both adenylate cyclase fragments. However, *E. coli* strains expressing at least one protein fusion containing ESAT-6T63A showed adenylate cyclase activity (**Figures 9** and **S6**), thus indicating protein-protein interaction. ESAT-6T63A interacted with itself and with ESAT-6 wild type (**Figures 9** and **S6**).

Altogether these results indicated that mutation in position 63 of ESAT-6 improved the self-association of ESAT-6, at least in the *in vivo* system used in this study.

## Discussion

The relevance of the innate immune response in TB disease has been addressed in several papers (36–38). Siddiqui et al. (39) reported the importance of a pre-established innate immune response to control bTB through vaccination trials in animal models and in humans. Recently, Netea et al. (40) have demonstrated that innate immunity can display adaptive characteristics after challenge with pathogens or their products. Also, in bTB a number of reports have demonstrated the central role of the innate immune response to control the intracellular replication of *M. bovis* in macrophages (8, 10). In particular, Carpenter et al. have described that PBMC from healthy cattle incubated with autologous BCG-infected bovine macrophages produced IFN-γ (41) and partially inhibited the intracellular multiplication of BCG (42). Also, a study in ferrets has suggested the cross talk between lymphocyte, NK and alveolar macrophages to control *M. bovis* intracellular replication (43). More recently, Denis et al. (44) and Endsley et al. (15) confirmed this finding in bTB. These two groups demonstrated that the control of the intracellular growth of *M. bovis* or *M. bovis* BCG relied on cell to cell contact between NK cells and infected macrophages (15, 44). In addition, Hamilton et al. described that NK cells isolated from young calves and co-cultured with BCG infected DCs displayed increased expression of CD25 and IFN-γ production (45).

All these findings highlight the multifaceted role of the innate immune system in response to bTB. Indeed, the granuloma formation, at least in its incipient stage, seems also to depend on the induction of the innate immune system by mycobacterial factors, as demonstrated in a zebrafish larva model of tuberculosis (46).

Despite these early reports, none of those studies have focused on the impact of the *M. bovis* strain variability in this immune response.

Here, we characterized the innate immune profile elicited by the virulent strain Mb04-303, which produces severe immunopathology in experimental infection models (20). The results showed that Mb04-303 induced a strong innate immune response, whereas Mb534 and MbNCTC10772 produced marginal or no response. Indeed, after *in vitro* infection of macrophages with Mb04-303, the innate immune response efficiently reduces mycobacterial replication.

Previous results from our group had shown that MbNCTC10772 did not produce pulmonary bTB in cattle (24) and Mb04-303 produced macroscopic lung lesions, with abundant cellular infiltration (20, 24). These findings indicate that innate immune response elicited by Mb04-303 would not be sufficient to control the disease *in vivo*. Probably, this strong innate response (reported in this study), together with the pro-inflammatory response (specific production of IFN-γ and IL-17) previously detected in experimentally infected cattle (47), is ultimately harmful to the host and facilitates the progression of bTB disease. Another plausible explanation to the poor protection observed in *M. bovis* 04-303 infections is that the bacteria develop strategies to evade the host immune response, as in *M. tuberculosis* infections (48).

Although the number of *M. bovis* strains here analyzed is limited, the results of this study suggest that virulent *M. bovis* strains are better immune stimulators than more attenuated *M. bovis* strains. However, Wedlock et al. have found that both attenuated and virulent *M. bovis* strains induced high levels of pro-inflammatory cytokine when inoculated in cattle and proliferated well in bovine alveolar macrophages (49). What remains to be determined is why a strong immune response cannot always efficiently control the bacterial replication and, therefore, the progress of the bTB disease.

Only strain Mb04-303 induced a low, but significant, cell activation of NKp46+ and WC1+ cells. These results are consistent with the induced expression of the key cytokines and the immune mediators described in the *Results* section.

Examples in the literature have reported activation of naïve cell proliferation in response to mycobacterial components. For example, bovine γδ T cells can proliferate and produce IFN-γ in response to protein and non-protein mycobacterial antigens following infection (50). Similarly, NK cells can also proliferate in contact with autologous macrophages infected with BCG (51). On the other hand, lipoarabinomanan/lipomanan molecules enhanced differentiation of naïve CD4 T human cells (52).

Although protective immune response against bTB relies on a Th1 cytokine profile and on one of its key player, IFN-γ, the role of this cytokine in activating naïve bovine macrophages against intracellular *M. bovis* growth is still unclear. The study of Aldwell et al. (53) has shown that IFN-γ pretreatment of bovine alveolar macrophages infected with *M. bovis* caused only partial growth inhibition (53), which is consistent with the low response of alveolar macrophages to IFN-γ (54). A previous study has shown that although incubation of NK cells with infected macrophages enhanced the levels of IFN-γ released by NK cell, this IFN-γ increase has no impact on controlling *M. bovis* intracellular replication (44). In this study, a gene transcriptional analysis of infected macrophages co-cultured with lymphocytes revealed that only strain Mb04-303 induced the expression of IFN-γ together with the expression of IL-22 and iNOS. Although the experimental design of this transcriptomic study did not allow us to distinguish which cell types differentially express IFN-γ, IL-22 or iNOS, we determined that NK cells, and to a lesser extend γδ T cells, were the main source of IFN-γ production in response to Mb04-303 infection. Indeed, the significant production of IFN-γ by γδ T and NK cells upon macrophage infection with Mb04-303, in relation to infection with the other *M. bovis* strains and the uninfected condition, strongly suggests that IFN-γ is at least in part responsible for controlling Mb04-303 replication.

Further research is necessary to define the contribution of IFN-γ to control *M. bovis* growth inside cells.

The lack of a commercial antibody against bovine IL-22 prevented us from defining the cell populations expressing this cytokine. Steinbach et al. have identified CD4+ and γδ T cells as the main producers of IL-22 in lymphocytes from *M. bovis*-infected cattle (55). On the other hand, Fu et al. have recently identified NK cells as a source of IL-22 (56).

Consistent with our findings, Magee et al. have shown that the expression of TLR2, TLR4 and MyD88 did not change in bovine macrophages after 6 h of infection with *M. bovis* in relation to the non-infected control (57). These authors, as well as Ma et al. (58), have reported that a MyD88-independent TLR signaling pathway is relevant in bTB.

The mannose cell receptors are key players in TB infection and this fact correlates with the high number of mannosylated proteins secreted by *M. tuberculosis* (59). Indeed, potential *M. tuberculosis* ligands of DC-SING have been previously reported (60). In this study, the expression of DC-SING was altered in co-cultures infected with Mb534. It has been proposed that DC-SING suppresses the pro-inflammatory responses against *M. tuberculosis* in human macrophages and that the inactivation of DC-SING renders human macrophages less permissive to *M. tuberculosis* replication (61). Also, it has demonstrated that a mutation of DC-SIGN, which resulted in its lower expression, protected against tuberculosis induced lung cavitation (62). Other study has linked the expression of DC-SIGN with TB disease progression (63). Therefore, the lower expression of DC-SING in co-cultures infected with Mb534 could explain in part the low persistence of this *M. bovis* strain inside macrophages and its reduced virulence in mice (21). Similarly, the attenuated vaccine strain, *M. bovis* BCG decreased the DC-SIGN expression on human DCs (64).

Another mannose receptor downregulated in co-cultures infected with Mb534 was MRC1. Some evidence support a beneficial role of MRC1 to control *M. tuberculosis* infections. For instance, Leisching et al. have reported that infection of murine macrophages with a *M. tuberculosis* Beijing clinical isolate induced downregulation of MRC1 gene expression, suggesting that the repression of MRC1 expression favor *M. tuberculosis* infection (65). In addition, two studies showed polymorphisms in MRC1 gene associated with susceptibility to TB in some human populations (66, 67). Other reports, by contrary, indicate that expression of MRC1 is detrimental for the host in the context of TB infections. Several studies have demonstrated that the binding of a mycobacterial glycolipid (MamLAM) to MRC1 impaired the phagosome fusion with lysosomes and further acidification (68). This last finding is consistent with the downregulation of DC-SING in Mb534 infection observed in this study and the fact that this strain has shown reduced capacity to avoid phagosome maturation in bovine macrophages (25).

Altogether the findings of our study suggest that the attenuated strain, Mb534, repressed the expression two mannose receptors and this could be associated with its reduced capacity of evading the cell immune response. However, cell infections with the virulent Mb 04-303 did not show the contrasting phenotype, which is upregulation of MRC1 and DC-SING. Therefore, more research is necessary to establish the importance of these mannose receptors in the interaction of *M. bovis* with the bovine cells.

In this study we compared the expression of the natural cytotoxicity receptor (NCR), NKp46, upon macrophage infections with the three *M. bovis* strains. This receptor, which was the first NCR described in NK cells, recognizes a spectrum of viral, bacterial (i.e. Vimentin from *M. tuberculosis*) and cell ligands, leading to NK cell activation. NKp46 also participates in the regulation of NK cell function (69). However, the role of NKp46 receptor in mediating immune response against TB is controversial.

A previous study described a direct relationship between the expression of NKp46 receptor and the mycobacterial killing by NK cells (70), whereas another study has reported a downregulation of this cytotoxic receptor in TB patients (71). In this last study, the treatment of patients resulted in a recovery of IFN-γ levels produced by NK cells, but failed to induce upregulation of the NKp46 receptor. Consistently with this latter finding, herein no direct relation was detected between IFN-γ induction and NKp46 expression in infected co-cultures. In fact, the opposite was evident: Mb04-303 infected co-cultures showed low levels of NKp46 receptor expression but the highest levels of IFN-γ production. On the other hand, NK cells of Mb534 infections registered the highest median values for NKp46 fluorescence intensity. Vankayalapati et al. have reported NKp46 overexpression in NK cells from healthy tuberculin reactors when the cells were co-cultured with autologous monocytes infected with the attenuated strain *M. tuberculosis* H37Ra (70). Therefore, the results of Vankayalapati et al.’s study and our results suggest that the expression and function of NKp46 depend on the mycobacterial virulence.

Another outcome of this study is that IFN-γ expression in Mb04-303 infected cells occurs in the presence of differentiated macrophages. This result emphasizes the relevance of mature APCs to achieve an effective innate immune response. Probably, certain immune mediators secreted from APCs and/or signals triggered during cell-to-cell contact interaction are decisive to mount this response. In line with this idea, Siddiqui et al. have detected IFN-γ secretion and expression of both CD25 and the major histocompatibility complex class (MHC) II on WC1+ γδ T cells co-cultured with *M. bovis*-infected DCs (8).

Live *M. bovis* BCG induced a more protective adaptive immune response than inactivated mycobacteria when tested as vaccines in cattle (72, 73); which suggests a central role for compounds secreted by viable mycobacteria in mediating this immune response. Consistently, our study demonstrated that *M. bovis* secreted compounds are crucial for the induction of an effector innate immune response in the context of mature APCs. In fact, NKp46+ and WC1+ populations showed increased levels of IFN-γ under both experimental conditions, infection with viable *M. bovis* and stimulation with lyophilized mycobacterial culture supernatant, whereas no response was detected with killed mycobacteria as stimulator.

Researchers have extensively studied the role of mycobacterial secreted lipids and proteins in modulating the immune response against TB. Prolonged exposure to MTB 19-kDa or LprA lipoprotein inhibits macrophage MHC-II expression and Ag processing by a TLR2-dependent mechanism and particularly the 19-kDa lipoprotein is an inhibitor of macrophage-responses to IFN-γ (74). Ishikawa et al. have also summarized the mycobacterial ligands and their wide variety of mammalian immune sensors, including the C-type lectin receptors (75). These authors proposed these receptors as intermediates between innate immune receptors and acquired immune receptors based on their abundance and broad ligand specificity. In addition, purified phosphatidylinositol mannoside molecules activate bovine lymphocyte populations, in particular NKT cells (76). Therefore, the highest activation of innate immune response upon stimulation with CS from Mb04-303 may be in part due to the highest secretion of immunogenic lipids, such as mycosid B, from this *M. bovis* strain.

A relevant outcome of this study is that the expression of ESAT-6T63A allele in Mb534 significantly increased the antigenicity of the recombinant *M. bovis* strain. Moreover, stimulation of co-cultures with CS from recombinant Mb534 expressing ESAT-6T63A also increased the percentage of NKp46+IFN+ cells.

ESAT-6 or EsxA is part of a mycobacterial protein family known as Esx. ESAT-6 forms a dimer/multimer with CFP-10 (culture filtrate antigen or EsxB) (Mb3904) and both proteins are strong stimulators of cell-mediated immunity (77). With the exception of *M. bovis* 04-303, the protein sequence of ESAT-6 is conserved in all sequenced *M. bovis* isolates (78).

We found that the amino-acid change in ESAT-6T63A improved the self-association of this protein, although the mutation maps outside of the known ESAT-6 protein interacting domain (79). Previous evidence indicated that in the absence of detergents, recombinant ESAT-6 self-associated to form dimers/multimers with specific functions (34). These protein dimers or multimers have shown to induce cell death, membrane pore formation and IFN-γ production, whereas treatment with a detergent disassociated ESAT-6 and abrogated these biological functions (34). Although in the present study we did not evaluate the impact of ESAT-6 mutation in the interaction with CFP-10, Refai et al. assessed the effects of dimer/multimer formation of ESAT-6 and the complex ESAT-6/CFP-10 on the infectivity of a recombinant BCG vaccine. They found that the dimers/multimers of ESAT-6 increased the infectivity of BCG in the same manner that the complex ESAT-6/CFP-10, suggesting that the dimers of ESAT-6 behave similarly to the physiological complex ESAT-6/CFP-10 (34).

Therefore, our and other findings suggest that the efficient innate immune response induced upon infection of bovine cell co-cultures with Mb04-303 is, at least in part, explained by the high protein-protein interaction capacity of ESAT-6T63A.

In conclusion, in this study we demonstrated that APCs play an essential role in the interaction of *M. bovis* with components of the innate immune response in the *in vitro* cell bovine system evaluated. In addition, the intensity of this innate immune response depends on the background of the *M. bovis* strains and certain unknown actively secreted mycobacterial compounds would be the main players in this host-pathogen interaction. Finally, the highly virulent strain Mb04-303 induces a powerful innate immune response capable of controlling *M. bovis* replication inside bovine macrophages and this immune response is at least in part explained by a non-synonymous mutation in *esxA* (*esat-6*) gene.

Future studies with *M. bovis* strains with varying degrees of virulence and *M. bovis* reference strains (AF2122/97 and AN5) will expand this work and give more information about the innate immune response towards *M. bovis* infection.

Finally, the findings in this study highlight the importance of evaluating the quality of the innate immune response induced by *M. bovis* and *M. tuberculosis* isolates before using them as parental strains for developing attenuated live vaccine candidates.

## Data Availability Statement

The original contributions presented in the study are included in the article/supplementary material. Further inquiries can be directed to the corresponding author.

## Ethics Statement

The animal study was reviewed and approved by CICUAE (Comité Institucional para el cuidado y uso de animales de experimentación), INTA.

## Author Contributions

The authors confirm contribution to the paper as follows: study conception and design: FB and FCB. Data collection: EAG, MJG, MMB, CM, MJ, and MM. Lipidomic analysis: MMB, CM, MJ and MM. Analysis and interpretation of results: FCB, MJG, and FB. Draft manuscript preparation: FB and FCB. All authors contributed to the article and approved the submitted version.

## Funding

This work was funded by ANCyPT Grants: PICT-2018 01113, PICT 2017-1721, PICT 2017-2704, INTA Grant I105 and I102. The funders had no role in study design, data collection and analysis, decision to publish, or preparation of the manuscript.

## Conflict of Interest

The authors declare that the research was conducted in the absence of any commercial or financial relationships that could be construed as a potential conflict of interest.

## Supplementary Material

Supplementary Figure 1 Representative flow cytometry using an anti-bovine CD14 antibody (MM61A, VMRD) for evaluation of macrophage purity in cultured adherent cells after 4-5 days of incubation.

Supplementary Figure 2 Representative dot plots. Gate strategy: (1) R1, lymphocyte gate used for WC1+ cells and (2) R2, gate used for NKp46+ cells. Dot plots from representative samples of WC1+IFN-γ+ in line A and NKp46+ IFN-γ+ in line B for non-infected and infected co-cultures (double positive events are represented in the upper right quadrant of each dot plot). Representative dot plots of NKp46+IFN-γ+ cells incubated with 5mg and 10mg lyophilized bacterial culture medium supernatant (CS) from Mb04-303 are depicted in line C.

Supplementary Figure 3 Cell viability evaluation for the mycobacterial growth inhibition assay in macrophages and co-cultures with autologous lymphocytes (+PBMC) infected with the different *M. bovis* strains **(A)** and for lymphocytes **(B)** stimulated with lyophilized bacterial culture media supernatant (CS).

Supplementary Figure 4 Lipid analysis of *M. bovis* strains **(A)** LC-MS analysis of secreted total lipids from *M. bovis* strains. **(B** and **C)** Thin-layer chromatograms of lipids from culture supernatants of *M. bovis* strains. Total lipids analyzed with a 9:1(v/v) mixture of n-hexane and diethyl ether **(B)** or 19:1 (v/v) mixture of chloroform and methanol **(C)** as the developing solvents. The arrows indicate the location of phthiocerol dimycocerosate **(B)** and mycoside **(B C)** as described in (22). 20 × 20 cm × 0.5-mm layers of silica gel G were used and chromatograms were sprayed with CuSO4 heated with heat gun.

Supplementary Figure 5 Western blot analysis in total secreted proteins from *M. bovis* strains with polyclonal α-ESAT-6. (1) Mb534::ESAT6 (2) Mb534::ESAT6T63A. Arrows indicate the position of ESAT-6 monomers and multimers.

Supplementary Figure 6
*In vivo* interaction of ESAT-6 alleles. Growth of co-transformed *E. coli* BTH101 strains in medium supplemented with maltose as sole carbon source. **(A)** significant difference between 1 and 2 (p<0.0001); **(B)** 1 and 3 (p<0.0001); **(C)** 1 and 4 (p<0.0001); **(D)** 2 and 3 (p<0.0001, p<0.05 at day 4); **(E)** 2 and 4 (p<0.0001) and **(F)** 3 and 4 (p<0.0001, p<0.001 at day 4).

Supplementary Table 1 Primers for real time PCR.
