# Supplementary figures and images for: Identifying Bacterial and Host Factors Involved in the Interaction of Mycobacterium bovis with the Bovine Innate Immune Cells
Source: Front Immunol. 2021 Jul 15;12:674643. doi: 10.3389/fimmu.2021.674643 (PMC8319915; doi:10.3389/fimmu.2021.674643)

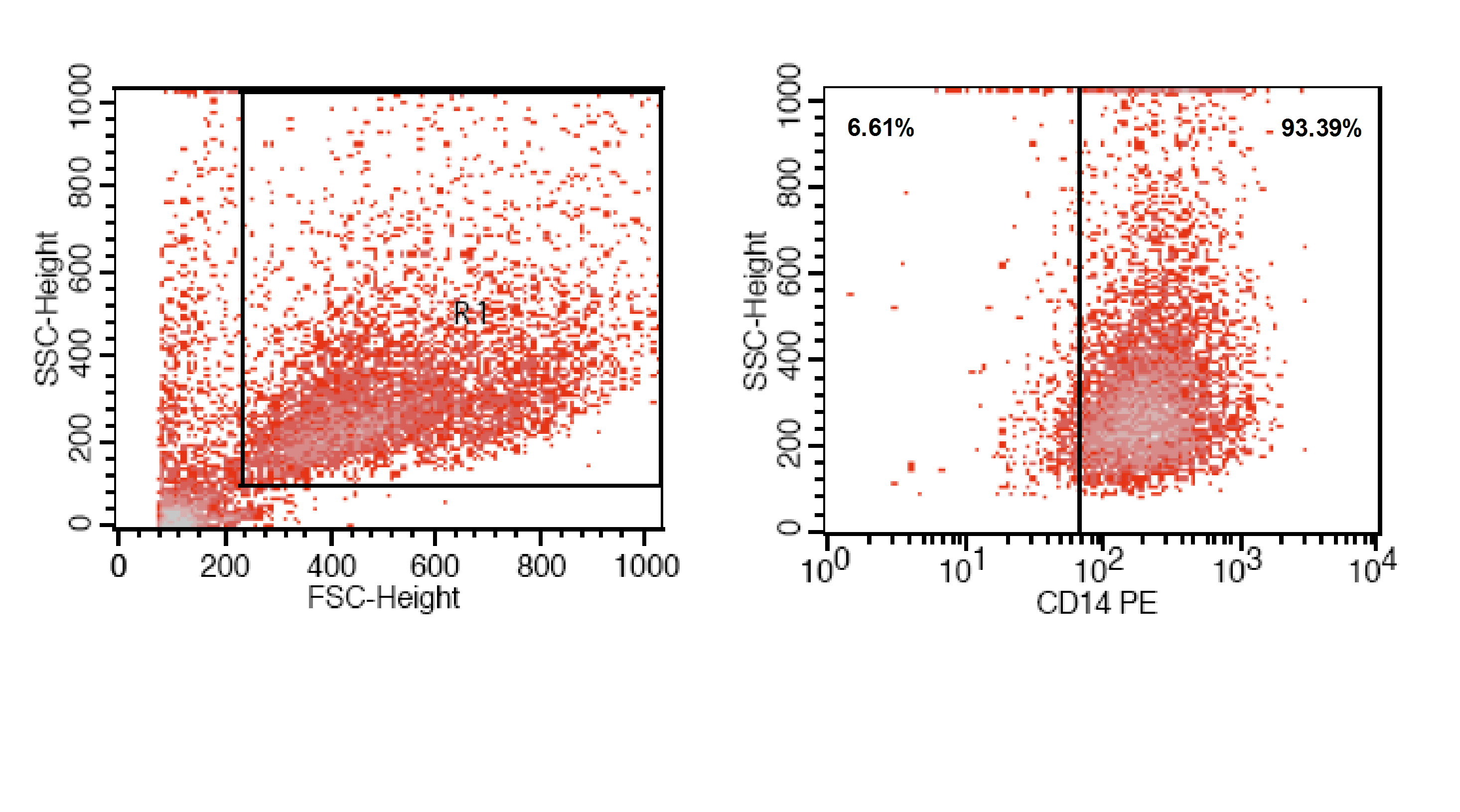

Supplement: Supplementary Figure 1 — Representative flow cytometry using an anti-bovine CD14 antibody (MM61A, VMRD) for evaluation of macrophage purity in cultured adherent cells after 4-5 days of incubation. [file Image_1.tif]

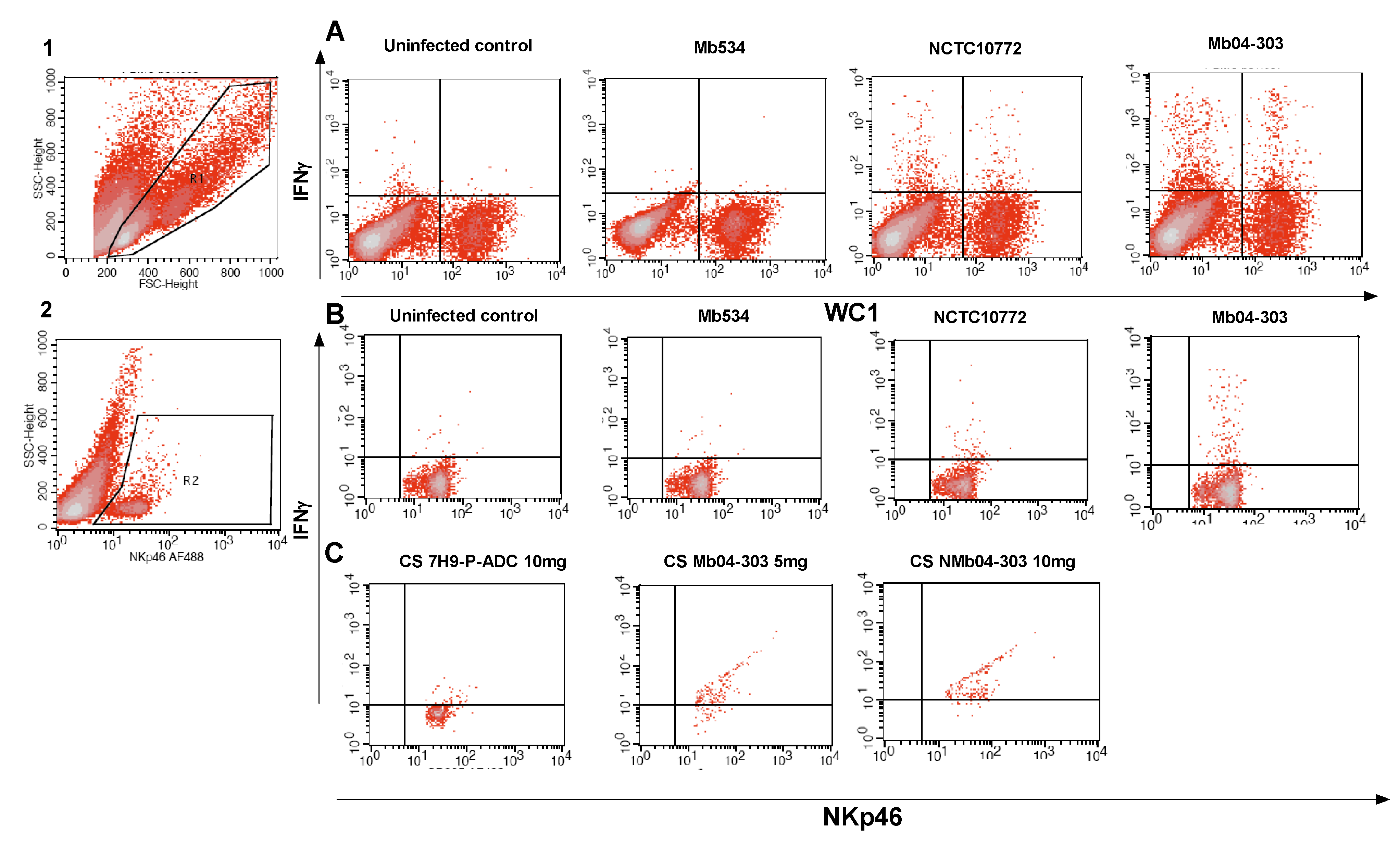

Supplement: Supplementary Figure 2 — Representative dot plots. Gate strategy: (1) R1, lymphocyte gate used for WC1+ cells and (2) R2, gate used for NKp46+ cells. Dot plots from representative samples of WC1+IFN-γ+ in line A and NKp46+ IFN-γ+ in line B for non-infected and infected co-cultures (double positive events are represented in the upper right quadrant of each dot plot). Representative dot plots of NKp46+IFN-γ+ cells incubated with 5mg and 10mg lyophilized bacterial culture medium supernatant (CS) from Mb04-303 are depicted in line C. [file Image_2.tif]

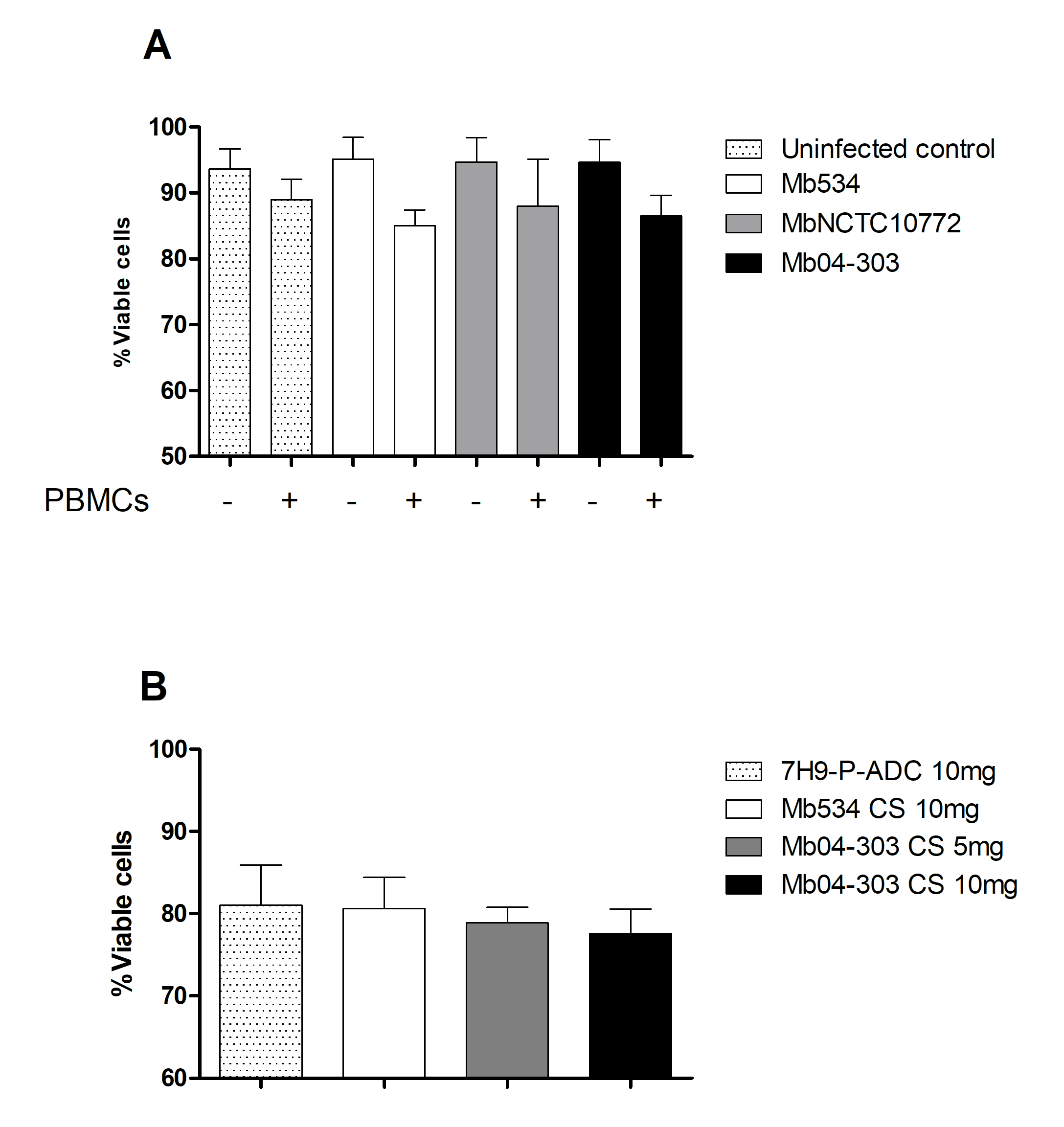

Supplement: Supplementary Figure 3 — Cell viability evaluation for the mycobacterial growth inhibition assay in macrophages and co-cultures with autologous lymphocytes (+PBMC) infected with the different M. bovis strains (A) and for lymphocytes (B) stimulated with lyophilized bacterial culture media supernatant (CS). [file Image_3.tif]

A

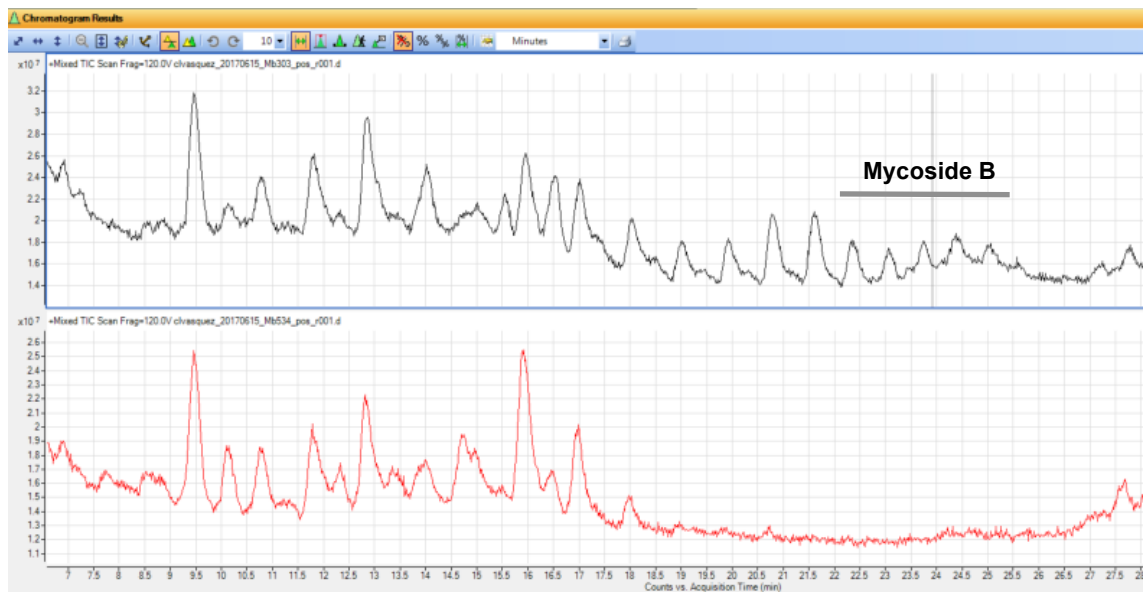

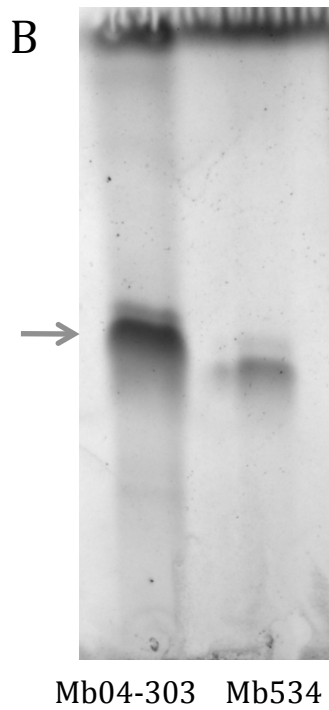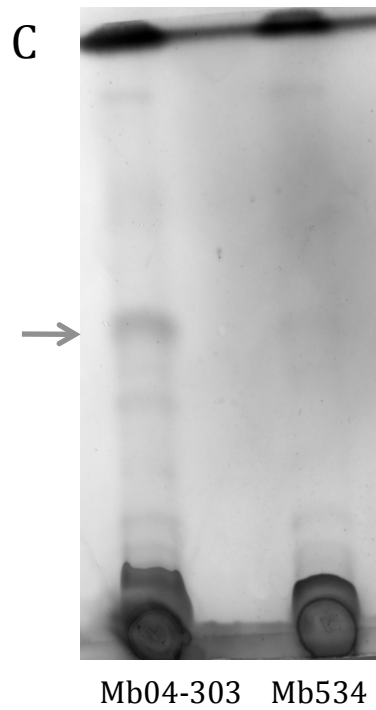

Supplement: Supplementary Figure 4 — Lipid analysis of M. bovis strains (A) LC-MS analysis of secreted total lipids from M. bovis strains. (B and C) Thin-layer chromatograms of lipids from culture supernatants of M. bovis strains. Total lipids analyzed with a 9:1(v/v) mixture of n-hexane and diethyl ether (B) or 19:1 (v/v) mixture of chloroform and methanol (C) as the developing solvents. The arrows indicate the location of phthiocerol dimycocerosate (B) and mycoside (B C) as described in (22). 20 × 20 cm × 0.5-mm layers of silica gel G were used and chromatograms were sprayed with CuSO4 heated with heat gun. [file Image_4.pdf]

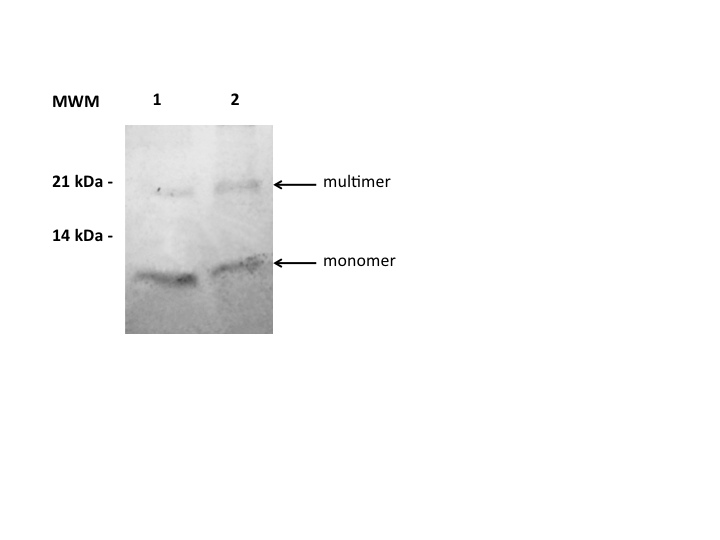

Supplement: Supplementary Figure 5 — Western blot analysis in total secreted proteins from M. bovis strains with polyclonal α-ESAT-6. (1) Mb534::ESAT6 (2) Mb534::ESAT6T63A. Arrows indicate the position of ESAT-6 monomers and multimers. [file Image_5.tif]

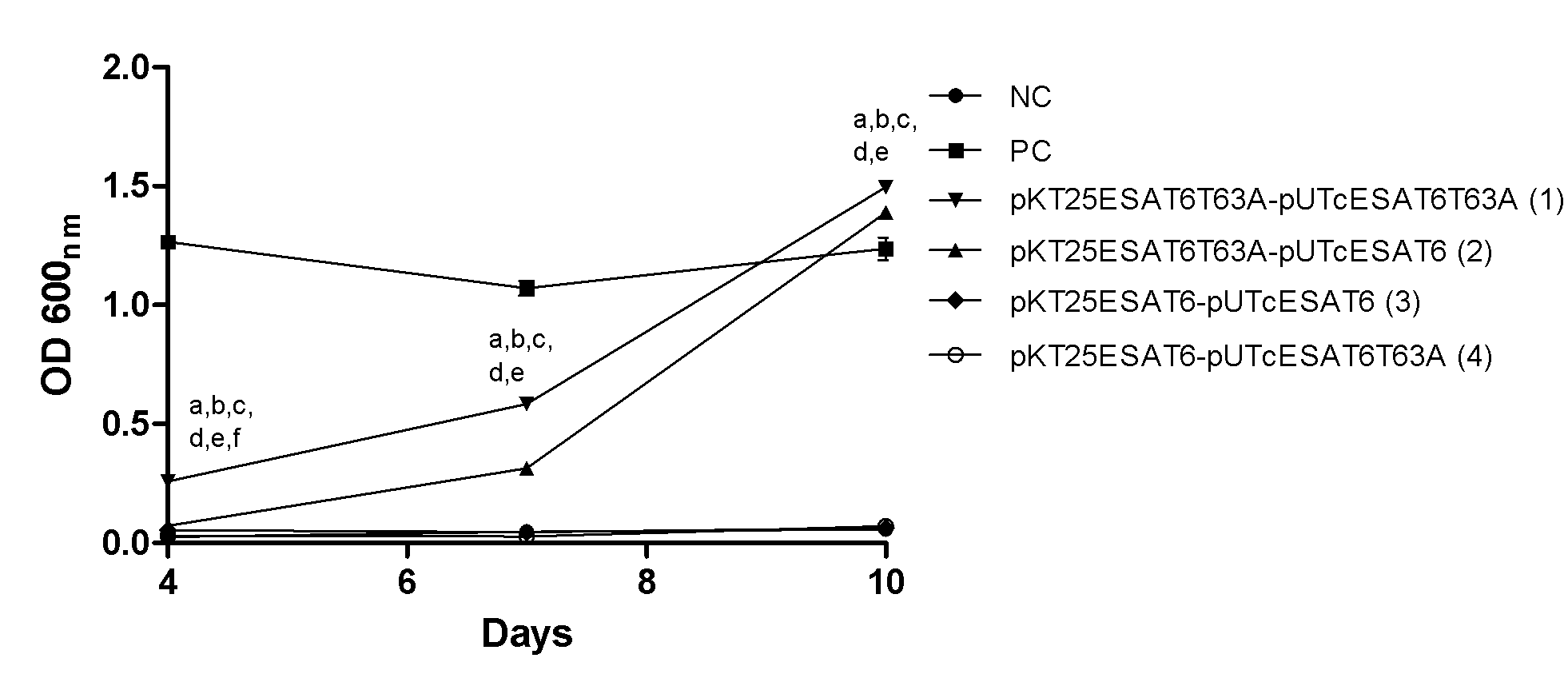

Supplement: Supplementary Figure 6 — In vivo interaction of ESAT-6 alleles. Growth of co-transformed E. coli BTH101 strains in medium supplemented with maltose as sole carbon source. (A) significant difference between 1 and 2 (p<0.0001); (B) 1 and 3 (p<0.0001); (C) 1 and 4 (p<0.0001); (D) 2 and 3 (p<0.0001, p<0.05 at day 4); (E) 2 and 4 (p<0.0001) and (F) 3 and 4 (p<0.0001, p<0.001 at day 4). [file Image_6.tif]
