# Supplementary material for: Identifying Bacterial and Host Factors Involved in the Interaction of Mycobacterium bovis with the Bovine Innate Immune Cells
Source: Front Immunol. 2021 Jul 15;12:674643. doi: 10.3389/fimmu.2021.674643 (PMC8319915; doi:10.3389/fimmu.2021.674643)
Supplement: Supplementary Table 1 — Primers for real time PCR. [file Table_1.doc]

Supplementary table 1: Primers for real time PCR

| *Primers sequences Amplicon Tm (ºC)* |
| --- |
| GAPDH  **F**: ATCTCTGCACCTTCTGCCGA 79.0  **R**: GCAGGAGGCATTGCTGACA  CXCL9 F: AAGGGATGATCCATCCAAAA 81.5  R: CAGGCTTCATTCCCATTCTT |
| IL-6 F: TGCTTGATCAGAACCACTGC 83.5  R: GCGATCTTTTGCTTCAGGAT  IL-10 F: GGAAGAGGTGATGCCACAGG 84.0  R: AGGGCAGAAAGCGATGACAG  iNOS F: AGAGCCTCTGGACCTCAACA 88.4  R: CTGCCCTCACAGGAGAGTTC  IFN-γ **F:** AGCTGATTCAAATTCCGGTGG 78.0 |
| **R:** GATTTTGGCGACAGGTCATTC |
| IL-17 **F:** GGACTCTCCACCGCAATGAG 81.0  **R**: GGTCCACCTTCCCTTCAGC  IL-22 F: CAAGGCTGGAAAATGGGTTA 84.0  R: GAAGCTAAGTCGGGGGTTTT  MHCII F: ACAGTGACCATCTCCCCATC 80.8  R: AACCACCGAACCTTGATCTG  MyD 88 F: CTGCAAAGCAAGGAATGTGA 85.4  R: TGCTGGGGAACTCTTTCTTC  TLR2 F: AACTCCATCCCCTCTGGTCT 86.0  R: TCAGGTTCACACACCTCTGC  TLR4 F: GGTTTCCACAAAAGCCGTAA 87.2  R: TCTGCAGGACGATGAAGATG  DC-SIGN F: TTCGTCTCACTGGGCTTCTT 87.1  R: CCTGGTGTCCAGCCACTTAT  MRC1 F: AGCACTCAGCATGGATTCCT 83.5  R: TTCAGCAGCACTTTCAATGG |
