# Supplementary material for: Identifying Bacterial and Host Factors Involved in the Interaction of Mycobacterium bovis with the Bovine Innate Immune Cells
Source: Front Immunol. 2021 Jul 15;12:674643. doi: 10.3389/fimmu.2021.674643 (PMC8319915; doi:10.3389/fimmu.2021.674643)
Supplement: Supplementary file 8 [file DataSheet_1.docx]

**Viability cell analysis:**

Viability was studied by flow cytometry (FACScalibur BD, 20,000 events recorded) using Fixable Viability Dye eFluor 660 (eBioscience).

**Lipid purification and analysis:**

Total lipids from culture supernatants were extracted following procedures described previously (24) and analysed by LC/MS as described by Sartain et al. (23). A high-resolution Agilent 6220 TOF mass spectrometer interfaced to a LC was used. Data files were analysed with Agilent’s Mass hunter workstation software (Version B.02.00, build 2.0.197.0) to identify compounds. The Agilent mass profiler program was used to compare lipids present in the sample. Most compounds were identified using the lipid database developed by Sartain et al. Compounds of interest were semi-quantified by comparing their relative abundance in the samples. The selected ion chromatograms indicated that both mycoside B and triglycerides are more abundant in strain 04-303; for mycoside B the ratio was approximately 470:1 (04-303:534). For triglycerides the ratio varied depending on the large of fatty acids, from 16:1 to 5.5:1 (04-303:534).

**Western blot analysis:**

Proteins from culture supernatants of bacterial cultures grown until stationary phase (30 days) were precipitated by incubation with 50% ammonium sulphate over night at 4 °C and centrifugation at 10,000 g. Protein pellets were resuspended in phosphate buffered saline (PBS) and dialyzed over night at 4 °C against PBS. The proteins were resuspended in a cracking buffer (60 mM Tris-Cl pH 6.8, 2% SDS, 10% glycerol, 2% β-mercaptoethanol, 0.01% bromophenol blue). Protein samples were subjected to electrophoresis in 15% SDS-PAGE gels, then transferred to nitrocellulose membranes. The membranes were blocked with TBS (10 mM tris-HClpH 7.5, 150 mM NaCl) supplemented with 5% skim milk for 30 min before incubating them with primary rabbit polyclonal antibody anti-ESAT-6 (BEI Resources) in a dilution 1/100 for 2 h. The nitrocellulose membranes were washed with TBS three times, and incubated with a secondary anti- rabbit alkaline phosphatase-conjugated antibody at a 1:10000 dilution for 2 h. Western blots were revealed by incubation with BCIP/NBT solution.

**Two hybrid assays (growth in maltose medium):**

*E. coli* BTH101 was cotransformed with a bait and prey recombinant plasmids. Co-transformed strains were grown in M63 medium supplemented with 0.3% maltose and antibiotics (ampicillin and kanamycin) at 30ºC with shaking. The bacterial growth was determined by OD600_nm_ measurements at 4, 7 and 10 days of culturing. Statistical analysis was performed using ANOVA and Bonferroni post test to compare the growth among the different co-transformed strains.
